# Rupatadine to prevent local allergic reactions to sublingual allergy immunotherapy: a case series

**DOI:** 10.1186/s13223-021-00630-6

**Published:** 2021-12-04

**Authors:** Anne K. Ellis, Lori Connors, Marie-Josee Francoeur, Douglas P. Mack

**Affiliations:** 1 Division of Allergy & Immunology, Department of Medicine, Queen’s University, 76 Stuart St, Kingston, ON K7L 2V7 Canada; 2 Department of Medicine, Dalhousie University, Halifax, NS Canada; 3 Division of Pediatric Allergy, University of Sherbrooke CISSS Montérégie Centre, Clinique des spécialistes Santé Dix 30, Elna Tiny Tots, Montréal, QC Canada; 4 Department of Medicine, McMaster University, Hamilton, ON Canada; 5 Halton Pediatric Allergy, Burlington, ON Canada

**Keywords:** Allergic rhinitis, Angioedema, H-1 antihistamine, Local allergic reactions, Platelet activating factor, Pruritus, Sublingual immunotherapy

## Abstract

**Background:**

Sublingual immunotherapy tablets (SLIT-T) are an effective treatment for allergic rhinitis (AR), but some patients experience local allergic reactions (LAR) in the first few weeks of treatment that can lead to treatment discontinuation. Although oral antihistamines are recommended for the treatment and pretreatment of LAR associated with SLIT-T, there are no clinical trial data to support this recommendation. Rupatadine is an H1 antihistamine that also inhibits platelet activating factor activity. The objective of this case series is to describe real-world clinical situations in which rupatadine was used to treat or mitigate SLIT-T–related LAR.

**Case presentations:**

Five cases are presented by the managing allergist and off-label use of rupatadine is their expert opinion only. Patients in all 5 cases were treated with a SLIT-T (e.g. ragweed, tree, grass, or house dust mites) for the management of allergic rhinitis and experienced bothersome LAR with the first SLIT-T administration. In 3 cases, rupatadine 10 mg was administered for the immediate treatment of LAR (either in-office with the first SLIT-T dose or for subsequent LAR experienced at home) and the symptoms resolved. In 3 cases, pretreatment with other second-generation H1 antihistamines was unable to prevent LAR and the patients discontinued the SLIT-T. In these 3 cases, switching to pretreatment with rupatadine allowed the patients to restart and tolerate SLIT-T treatment with minimal or no LAR. In these patients with an established history of LAR, proactive pretreatment with rupatadine in subsequent seasons or with initiation of a different SLIT-T mitigated the previously experienced LARs.

**Conclusions:**

In the cases presented, treatment with rupatadine resolved LAR associated with SLIT-T treatment and rupatadine pretreatment appeared to mitigate subsequent LAR. Rupatadine may be an option to treat or improve the tolerability of the SLIT-T, potentially improving early treatment persistence.

## Background

Allergy immunotherapy (AIT) is a recommended and effective treatment for allergic rhinitis (AR). AIT can be administered in the form of sublingual immunotherapy tablets (SLIT-T), the safety and tolerability of which have been demonstrated in multiple clinical trials [1–5] However, many patients experience mild to moderate local allergic reactions (LAR) to the SLIT-T; more than 80% of the adverse events related to SLIT-T treatment in clinical trials are LAR [6, 7]. The most common LAR with SLIT-T are throat irritation, oral pruritus, ear pruritus, tongue pruritus, and mouth edema [6, 7]. Data from clinical trials indicate that the LAR associated with SLIT-T generally resolve within 30 to 60 min and cease after 2 weeks [6, 8]. In most cases, the LAR do not require medical intervention, but for some patients the LAR may be uncomfortable and lead to SLIT-T discontinuation early in treatment [6, 9, 10].

**Fig. 1:**
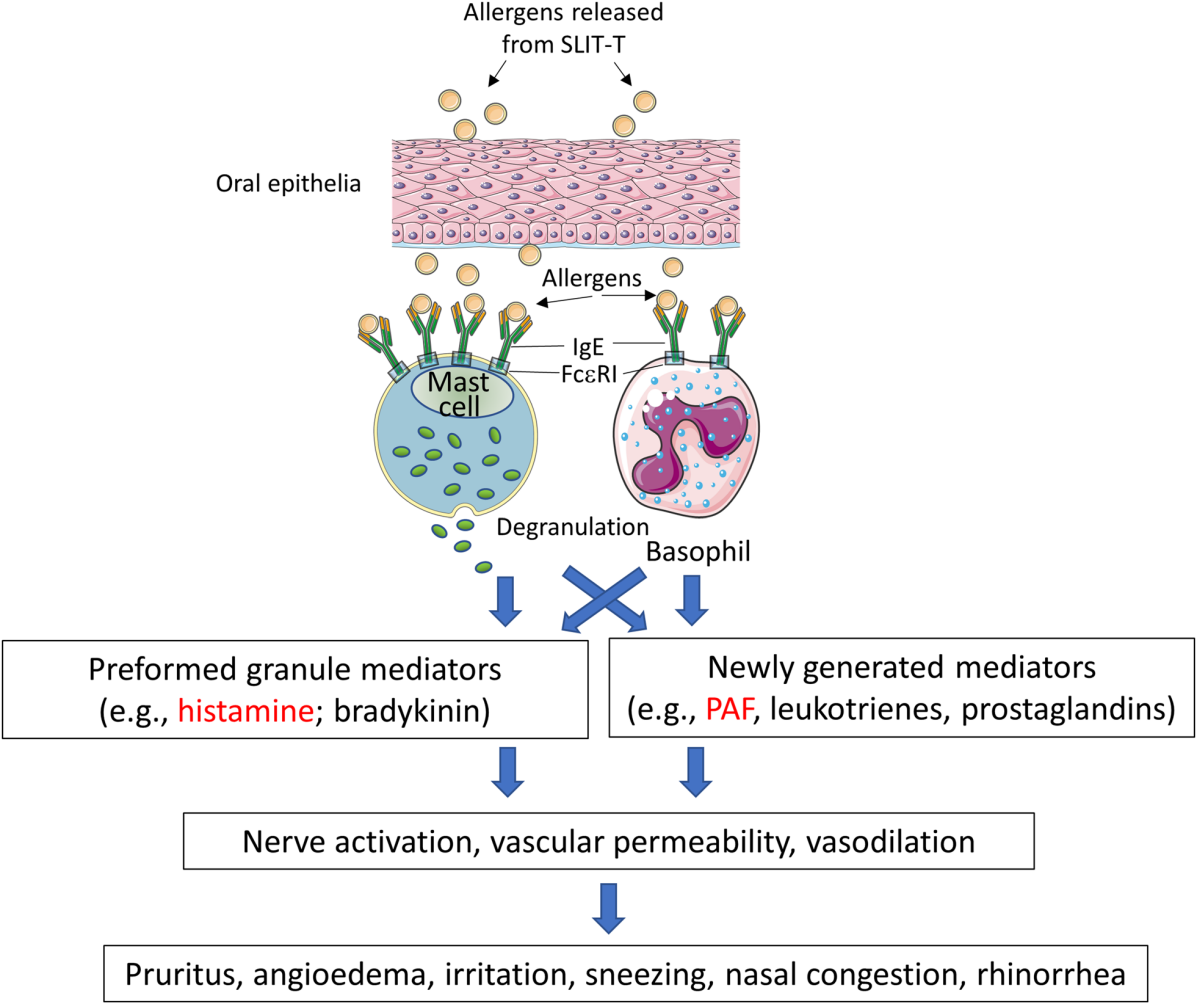
Presumed pathophysiology of local allergic reactions associated with sublingual immunotherapy tablets (SLIT-T)

The LAR induced by SLIT-T are generally acute allergic reactions similar to skin prick test or pollen food syndrome reactions [6, 11]. The allergen introduced into the oral mucosa elicits an IgE-mediated acute allergic response, in which histamine, platelet activating factor (PAF), and other mediators play a role (Fig. 1). Allergy expert panels suggest that H1 antihistamines can be used to treat or prevent LAR associated with SLIT-T [9, 10]. However, no second-generation H1 antihistamines are approved for this indication, and no clinical trials have formally evaluated the impact of antihistamines on SLIT-T LAR. Rupatadine is a second-generation H1 antihistamine that is approved in Canada for the treatment of AR and chronic spontaneous urticaria in patients age 2 years and older [12]. Unlike other H1 antihistamines, rupatadine has potent anti-PAF activity through specific inhibition of the PAF receptor [13, 14]. The objective of this case series is to describe real-world clinical situations in which rupatadine was used to treat or mitigate SLIT-T–related LAR.

## Case presentations

The cases are presented by the managing allergist and off-label use of rupatadine is used at the discretion of the same. The consent form, protocol, and data collection sheet were reviewed for ethical compliance by the Queen’s University Health Sciences and Affiliated Teaching Hospitals Research Ethics Board and ethical clearance was granted.

Verbal and written consent was obtained from each case patient, when possible. When consent could not be obtained, the information in the case was presented in such a way as to anonymise the patient. Written consent for publication of the patient photograph was obtained from the patient’s guardian.

### Case 1

A 9-year old male presented with a history of AR during grass pollen season. The patient had concomitant controlled asthma treated with a daily low-dose inhaled corticosteroid (ICS). Skin prick test (SPT) indicated a sensitivity to grass (wheal = 9 mm) and tree mix (wheal = 7 mm).

A pre- and co-seasonal regimen of the 5-grass SLIT-T was prescribed. Beginning on day 1 during in-office administration, the patient experienced substantial, distressing daily mouth and throat symptoms that lasted up to 60 min after SLIT-T administration. Mild ear pruritus was also noted. Moderate lip angioedema occurred for the first 2 days of treatment, along with mild sublingual cavity angioedema. The patient was premedicated with 5 mg oral cetirizine before subsequent SLIT-T administrations but discontinued the SLIT-T after 5 days because of the substantial daily mouth and throat symptoms. A sublingual sensation of possible mild swelling was noted for a few days after discontinuing the SLIT-T.

Treatment with the 5-grass SLIT-T was restarted one month after discontinuing initial treatment. Beginning on day 1 of the SLIT-T restart, pretreatment with rupatadine 5 mg liquid solution was given 2 h before each SLIT-T dose. The patient experienced mild palate pruritus that lasted for less than 10 min on day 1 of the SLIT-T restart and mild ear pruritus that lasted for less than 10 min on day 3. No angioedema or sublingual symptoms occurred. Pretreatment with rupatadine was given for the first 2 weeks of the SLIT-T restart and then was discontinued with no subsequent LAR associated with the SLIT-T. When the 5-grass SLIT-T was initiated for the next year’s grass pollen season, the same rupatadine pretreatment regimen was proactively used for the first week of the SLIT-T administration. No LAR associated with the SLIT-T occurred. There were no adverse events associated with the rupatadine treatment.

### Case 2

A 15-year old female presented with a history of AR during ragweed pollen season. SPT indicated a sensitivity to ragweed (wheal = 14 mm), *Dermatophagoides pteronyssinus* (*D. pteronyssinus*; wheal = 8 mm), and *Dermatophagoides farinae* (*D. farinae*; wheal = 6 mm).

A pre- and co-seasonal regimen of the ragweed SLIT-T was prescribed. Over the first 7 days of SLIT-T administration, the patient experienced substantial and escalating mouth and throat symptoms. A globus sensation in the throat occurred on 3 of the first 7 days of SLIT-T treatment and a visible uvular swelling (observed in the office) occurred on 2 days. Sneezing was noted on 2 days. Daily pretreatment with desloratadine was begun on day 2 of SLIT-T treatment. However, on the 7th day of SLIT-T treatment a mild cough and throat symptoms developed and the patient discontinued the SLIT-T.

Treatment with the ragweed SLIT-T was restarted 6 weeks after discontinuing initial treatment. Beginning on day 1 of the SLIT-T restart, pretreatment with rupatadine 10 mg tablet was given 2 h before each SLIT-T dose. The patient experienced mild mouth pruritus for the first 2 days of treatment. Pretreatment with rupatadine was given for the first 9 days of the SLIT-T restart and then was discontinued. The patient successfully completed the seasonal course of ragweed SLIT-T with no subsequent LAR and with a good symptom response. There were no adverse events associated with the rupatadine treatment.

### Case 3

A 54-year old female presented with seasonal and perennial AR. Allergy history was assessed via telemedicine because of the COVID-19 pandemic. The patient had asthma triggered by cat exposure, which the patient avoided and therefore did not need asthma controller medication. Serum IgE results indicated sensitivity to birch, ragweed, grass mix, cat dander, dog dander, *D. pteronyssinus*, and *D. farinae*.

A pre- and co-seasonal regimen of the tree and timothy grass SLIT-T was prescribed, with plans for ragweed and house dust mite (HDM) SLIT-T to follow. Within 5 min of the first tree SLIT-T administration in the office, the patient experienced distressing mouth pruritus. Rupatadine 10 mg tablet was administered immediately and the pruritus resolved within 25 min. For subsequent home administration, the patient was instructed to take rupatadine 10 mg every evening before bed for 2 weeks and to take the SLIT-T every morning. When the patient returned to the office 1-month later to initiate the timothy grass SLIT-T, she reported that the mouth pruritus associated with the tree SLIT-T had completely resolved with no recurrence during home administration. She had discontinued the rupatadine pretreatment. Proactive pretreatment with rupatadine 10 mg tablet was given 1 h before the first timothy grass SLIT-T dose and the patient experienced no LAR. The patient continued to pretreat with rupatadine 10 mg tablet for the first 2 weeks of the timothy grass SLIT-T as a precaution. There were no adverse events associated with the rupatadine treatment.

### Case 4

A 17-year old male presented with a history of severe AR during the ragweed pollen season. The patient also had mild exercise-induced asthma which was treated with a short-acting beta-agonist as needed. SPT indicated sensitivity to ragweed (wheal = 12 mm).

**Fig. 2:**
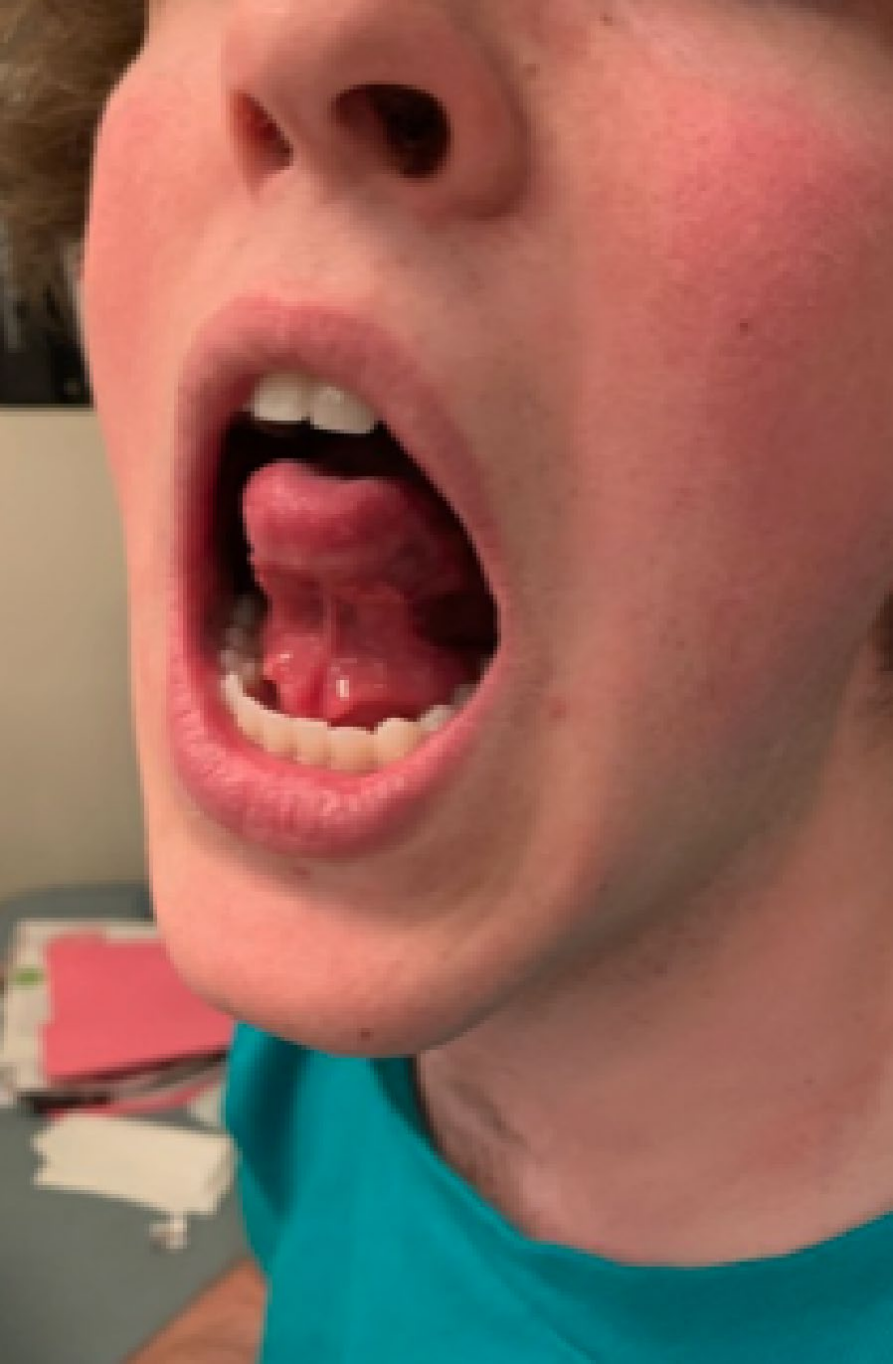
Sublingual swellings associated with the ragweed SLIT-T

A pre- and co-seasonal regimen of the ragweed SLIT-T was prescribed. With the first dose of the ragweed SLIT-T in the office, the patient experienced intense tongue, mouth, and palate pruritus. Cetirizine 10 mg liquid solution was administered immediately and the patient was kept under observation for 50 min. The LARs resolved and the patient was sent home with instructions to administer cetirizine 10 mg 1 h before daily at-home administration of the ragweed SLIT-T. After 1 week of ragweed SLIT-T treatment and cetirizine pretreatment, the patient was still experiencing mouth pruritus but was highly motivated to continue the ragweed SLIT-T treatment. Premedication was switched to rupatadine 10 mg tablet and the ragweed SLIT-T was cut in half for 7 days. After finishing the 7 days of rupatadine pretreatment and half-dose of the ragweed SLIT-T, the patient reinitiated the full-dose SLIT-T with cetirizine pretreatment (rupatadine was not used because it was not reimbursed by the patient’s insurance company; rupatadine was reimbursed during subsequent seasons). Within 3 days, the patient reported intense mouth pruritus and 2 large sublingual swellings (Fig. 2). Treatment with rupatadine 10 mg liquid solution was administered and the sublingual swellings regressed after a few hours. The patient then discontinued SLIT-T treatment. Once the ragweed pollen season started, the patient was experiencing severe AR symptoms despite daily oral antihistamine and intranasal corticosteroid treatment.

The following May, pre-seasonal treatment with the ragweed SLIT-T was proactively initiated along with pretreatment with rupatadine 10 mg tablet a few days before and 1 h before the first SLIT-T dose in the office. The patient experienced only mild mouth pruritus with the first dose of the SLIT-T. Pretreatment with rupatadine 10 mg tablet 1 h before SLIT-T administration was maintained for the first 2 weeks, then the pretreatment rupatadine tablet dose was cut to 5 mg for an additional 2 weeks before pretreatment was totally discontinued. There were no subsequent LAR associated with the SLIT-T and the patient successfully completed the seasonal course of ragweed SLIT-T. There were no adverse events associated with the rupatadine treatment.

### Case 5

A 28-year old male presented with perennial ARC. The patient had concomitant mild asthma well controlled by a combination ICS/long-acting beta-agonist. SPT indicated sensitivity to grass mix (wheal = 7 mm) and HDM (wheal for *D. farinae* = 6 mm and for *D. pteryonyssinus* = 8 mm).

A regimen of daily HDM SLIT-T and pre- and co-seasonal timothy grass SLIT-T was prescribed. The patient tolerated initiation with the timothy grass SLIT-T well, but with the first dose of the HDM SLIT-T in the office the patient experienced intense mouth pruritus and mild tongue angioedema. Rupatadine 10 mg tablet was administered immediately and the LARs resolved within 40 min. For subsequent home administration, the patient was instructed to take rupatadine 10 mg tablet 30 min before SLIT-T administration for 8 weeks. After 8 weeks, the rupatadine pretreatment was reduced to every other day and then ultimately discontinued. The rupatadine pretreatment improved the tolerability of the HDM SLIT-T and the patient experienced only mild mouth pruritus with no angioedema. There were no adverse events associated with the rupatadine treatment.

## Discussion and conclusions

In the 5 cases presented, treatment with rupatadine resolved LAR associated with SLIT-T treatment and rupatadine pretreatment appeared to mitigate subsequent LAR. These cases are the first published reports focusing on the impact of an antihistamine for SLIT-related LAR treatment or pretreatment.

The ability to mitigate or prevent LAR is important for early persistence with SLIT-T treatment. In a study of 252 patients who initiated SLIT, 8% discontinued because of side effects in the first 10 days of treatment [2]. Before starting SLIT-T treatment, patients should be educated about the potential for LAR, what they may feel like, how long they will last, and if they will continue to reoccur [6]. LAR typically stop recurring after about 2 weeks, [6, 8] and pretreatment with antihistamines may enable the patient to better tolerate the LAR and continue treatment until then.

Proactive pretreatment with rupatadine was used in some of the presented cases to prevent LAR with SLIT-T restart in subsequent seasons or with initiation of a different SLIT-T. A history of LAR to SLIT-T was already established in these patients. An expert panel has recommended that the first administration of a SLIT-T should not be pretreated with antihistamine so that the patient’s reaction to the SLIT-T can be properly assessed by the managing allergist [9].

Treatment of the LARs was sometimes with a liquid solution of antihistamine rather than a tablet. In our experience, the liquid solution is advantageous in relieving patient anxiety because the patient often believes the liquid works faster. Furthermore, the liquid solution coats the oral mucosa and may therefore induce a quicker response to the LAR than a tablet formulation, although this has not been scientifically evaluated. An analogous situation is allergic conjunctivitis where ophthalamic antihistamine formulations have an onset of action of about 15 min compared with 1–2 h for tablet antihistamine formulations [15].

In 3 of the cases, pretreatment with other second-generation H1 antihistamines was implemented to prevent LAR, but the LAR continued and were bothersome enough that the patients chose to discontinue the SLIT-T. In all 3 cases, the SLIT-T treatment was restarted with a rupatadine pretreatment and the LAR were minimal or absent compared with the previous SLIT-T treatment period. There are a couple of potential explanations as to why the LAR appeared to be reduced with rupatadine pretreatment but not the other antihistamines. First, rupatadine is the only H1 antihistamine that has anti-PAF effects through specific inhibition of the PAF receptor [13, 14]. Rupatadine may have inhibited PAF-induced LAR pathophysiologic processes in addition to those related to histamine, although a specific role for PAF in SLIT-T–associated LAR pathogenesis has not been elucidated. Indirect evidence for the role of PAF in SLIT-T–associated LARs is based on the known active role of PAF in hypersensitivity reactions and allergic inflammation, as well as the ability of anti-PAF agents to ameliorate these effects [16–18]. Secondly, there is a natural decrease in LAR over time with SLIT-T treatment as allergen tolerance develops. Therefore, the mitigation in LAR observed with rupatadine pretreatment could be attributed to the development of some level of tolerance from the first SLIT-T treatment period rather than a specific rupatadine effect. However, in some of the cases there was a substantial gap in taking the SLIT-T so tolerance may have been limited.

A limitation of these case observations is that while rupatadine treatment appeared to mitigate LAR in these particular patients, the results cannot be extrapolated to other patients and data from clinical trials are needed.

LAR associated with SLIT-T treatment can be uncomfortable for some patients and lead to treatment discontinuation. The presented cases demonstrate that rupatadine may be an option to treat or improve the tolerability of the SLIT-T, potentially improving early treatment persistence.

## Data Availability

All data supporting the conclusions of this article are included within the article.

## Abbreviations

AIT: Allergy immunotherapy
AR: Allergic rhinitis
HDM: House dust mite
ICS: Inhaled corticosteroid
LAR: Local allergic reactions
PAF: Platelet activating factor
SLIT: Sublingual immunotherapy
